# Comparison of Biological and Genetic Characteristics between Two Most Common Broad-Leaved Weeds in Paddy Fields: *Ammannia arenaria* and *A. multiflora* (Lythraceae)

**DOI:** 10.3390/biology12070936

**Published:** 2023-06-30

**Authors:** Yuan Gao, Shenghui Li, Guohui Yuan, Jiapeng Fang, Guohui Shen, Zhihui Tian

**Affiliations:** 1 Eco-Environmental Protection Research Institute, Shanghai Academy of Agricultural Sciences, Shanghai 201403, China; gaoyuan@saas.sh.cn (Y.G.); yuanguohui@saas.sh.cn (G.Y.); fangjiapeng@saas.sh.cn (J.F.); 2 College of Agriculture, Anshun University, Anshun 561000, China; lishenghui202304@163.com

**Keywords:** *Ammannia*, chloroplast genome, single nucleotide polymorphisms, repeat, phylogenetic tree

## Abstract

**Simple Summary:**

*Ammannia arenaria* and *A. multifloras*, the most common broad-leaved weeds in rice paddy fields in China, are morphologically similar at the seedling stage. However, their degree of damage to rice may vary. Furthermore, the sensitivity of two species to the constantly emerging new herbicides is also unknown. This study conducted field investigations, indoor biological experiments, and chloroplast genome construction and analysis to clarify the differences in biological characteristics, herbicide sensitivity, and chloroplast genetics between *A. arenaria* and *A. multifloras*. Our research results may provide theoretical basis for weed occurrence prediction, selection of herbicides, and *Ammannia* classification and distinction. Furthermore, the results provided valuable biological information on cp genomes of *Ammannia* that will be useful to identify and classify *Ammannia*, and study their phylogenetic relationships and evolution.

**Abstract:**

*Ammannia arenaria* and *A. multifloras*, morphologically similar at the seedling stage, are the most common broad-leaved weeds in paddy fields. Our study showed that *A. arenaria* occupied more space than *A. multifloras* when competing with rice. However, *A. multifloras* germination has lower temperature adaptability. No difference in sensitivity to common herbicides between two *Ammannia* species was observed. Chloroplast (cp) genomes could be conducive to clarify their genetic relationship. The complete cp genome sequences of *A. arenaria* (158,401 bp) and *A. multiflora* (157,900 bp) were assembled for the first time. In *A. arenaria*, there were 91 simple sequence repeats, 115 long repeats, and 86 protein-encoding genes, one, sixteen, and thirty more than those in *A. multiflora*. Inverted repeats regions expansion and contraction and the phylogenetic tree based on cp genomes demonstrated the closely relationship between the two species. However, in *A. arenaria*, 20 single nucleotide polymorphisms in the CDS region were detected compared to *A. multiflora*, which can be used to distinguish the two species. Moreover, there was one unique gene, *infA*, only in *A. arenaria*. This study provides reliable molecular resources for future research focusing on the infrageneric taxa identification, phylogenetic resolution, population structure, and biodiversity of *Ammannia* species.

## 1. Introduction

*Ammannia* belongs to the annual herbs of the Lythraceae, containing 25 species, mainly distributed in humid areas such as marshes, waters, or paddy fields worldwide [1]. The genus comprises upright and solid stems extending approximately 150 cm high, flowers have four petals, and seeds are inverted pyramidal; one side is round, and the other is irregularly concave [2]. This genus is the most common broad-leaved weed in rice fields and is a successful competitor for nutrition and space during the rice growth period [3,4,5]. The most common *Ammannia* weeds in China’s paddy fields are *A. arenaria* and *A. multiflora* [5,6,7]. These two weeds have very a strong ability to adapt to the environment and can grow under water in the early stage of rice planting and complete vegetative growth and reproduce, provided the soil is maintained moist in the later stage. Although *A. arenaria* and *A. multiflora* seeds are very small, their quantity is very large, making them the most problematic in rice fields [5,6].

Managing *A. arenaria* and *A. multiflora* in paddy fields is a considerable challenge in crop protection. Because the seedbanks of this genus are very large [5,6], the use of pre-emergence herbicides to control it is necessary [8]. The sulfonylureas herbicide, bensulfuron-methyl, which belongs to the acetolactate synthase (ALS) inhibitors [9], was most frequently used to control *A. arenaria* and *A. multiflora* [10,11,12]. However, such herbicides acting on a single target-site are prone to develop resistance [13,14]. Unfortunately, bensulfuron-methyl resistance has been reported in *A. arenaria* [15,16] and *A. multiflora* [17,18]. The failure of weed control at pre-emergence inevitably strengthens post-emergence control. The harmfulness of the two weeds is different. Generally, adult *A. arenaria* is taller than rice and requires more space and nutrients, whereas adult *A. multiflora* is smaller [19]; therefore, the focus of management should be inclined. However, before flowering, *A. arenaria* and *A. multiflora* are difficult to distinguish. Presently, chloroplast (cp) genomes sequencing and identifying their genetic lines contribute to distinguishing two *Ammannia* weeds, establishing a basis for scientific management. From a different perspective, *A. arenaria* and *A. multiflora* are important medicinal plants [20], which is helpful for the treatment of many diseases, including otitis media [21] and thyroid nodules [22]. Chloroplast genome information also facilitates the utilization of *Ammannia* resources and exploitation of allied species.

Chloroplasts (cp) are organelles in photosynthetic plants or algae that perform photosynthesis [23]. Chloroplasts contain genetic material, and their genomes are highly conserved owing to the lack of recombination, haploidy, and uniparental inheritance. Fundamentally, they can provide rich evolutionary information [24,25,26]. In addition, the cp genome is small and easy to obtain completely compared to the nuclear genome; therefore, it has unique research value in phylogeny, species identification, and population genetics [27]. Because of these characteristic properties, determining and analyzing ribosome organization in the cp system has become an important mechanism for addressing plant phylogeny and assessing biodiversity. Generally, the cp genome has a typical quadripartite structure [28,29,30] and its circular structure is organized into large single-copy (LSC) and small single-copy (SSC) regions, separated by inverted repeats (IRs), which are a pair of sequences with opposite orientations, named IRa and IRb [24,31,32]. Sequences between IRa and IRb regions can generate triggered flip-flop recombination, stabilizing single-copy regions [33]. The cp genome is particularly useful for studies characterizing the phylogeny and history of most plant lineages in the context of reticular-type evolution (hybridization) and polyploidy [34,35,36]. With the advancement of the cp genome-sequencing technology and in-depth understanding of the cp genome by researchers, the genetic relationship of multiple genera, such as *Camellia*, *Taxodium*, and *Pterocarpus*, have been uncovered [27,33,37]. To date, information about the composition, structure, and differences between species, as well as the evolutionary relationships of *Ammannia* species based on cp genome is still limited.

This study aimed to explore a complete analysis and comparison of germination conditions, field morphology, herbicides sensitivity, and cp genomes of *Ammannia* species, *A. arenaria* and *A. multifloras* (Supplementary Figure S1) collected in paddy fields for the first time, and provide knowledge for the identification of the two morphologically similar plants. Therefore, this study also provides a theoretical basis for the regeneration of diversity and resource utilization of this genus.

## 2. Materials and Methods

### 2.1. Comparison of Morphology and Seed Germination Conditions

The plant height and maximum lateral distance of *A. arenaria* and *A. multiflora* in rice fields in Shanghai were measured in November 2022. More than 20 plants were randomly selected; however, no more than three plants were sampled from each paddy field. The seeds of three populations per species were collected (Table 1), dried to constant weight, and weighed. A total of 294 rice fields were investigated and fields with *A. arenaria* and *A. multiflora* were recorded, and the frequency was calculated. The culture dish method was used to test the germination conditions of the two species. The test temperature was set to 15, 20, 25, 30, and 35 °C, the pH was set to 3.0, 5.0, 7.0, 9.0, and 11.0, and the osmolarity was set to −0.06, −0.17, −0.32, −0.53, and −0.79 MPa (mass fraction of PEG6000 was 5%, 10%, 15%, 20%, and 25% [38]). The number of germinated seeds was investigated and recorded every other day until no new seeds germinated. Each experimental treatment contained three biological replicates and the experiment was conducted twice.

Significant differences in seed germination rate of *A. arenaria* and *A. multiflora* were compared using Duncan’s multiple range test (*p* < 0.05). Analysis of variance (ANOVA) was performed using the SPSS Statistics (for Windows, Version 20.0. Armonk, NY, USA: IBM Corp.). Significant differences in the plant height, maximum lateral distance, seeds weight, and frequency of weed occurrence was also subjected to significance analysis with the use of SPSS Statistics (for Windows, Version 20.0. Armonk, NY, USA: IBM Corp.) by a Student’s *t*-test (*p* ≤ 0.05).

### 2.2. Determination of Sensitivity to Common Herbicides

The stems and leaves of three *A. arenaria* or *A. multifloras* populations (Table 1) were sprayed with pyrazosulfuron-ethyl (Liben Crop Science, Lianyungang, Jiangsu Province, China), pyraquinate (Shandong CYNDA (Chemical) Co., Ltd., Jinan, Shandong Province, China), florpyrauxifen-benzyl (Corteva Agriscience, Wilmington, DE, USA), and 2-methyl-4-chlorophenoxyacetic acid, sodium salt (MCPA-Na, Jiangsu Jian Gu Chemical industry Co., Ltd., Suqian, Jiangsu Province, China) when the plants reached the 5–6-leaf stage using a 3WP-2000 walking-type spraying system (Nanjing, China). The final doses were 1.875, 3.75, 7.5, 15, 30, and 60 g a.i. ha^−1^ for pyrazosulfuron-ethyl, 4.6875, 9.375, 18.75, 37.5, 75, and 150 g a.i. ha^−1^ for pyraquinate, 0.5625, 1.125, 2.25, 4.5, 9, and 18 g a.i. ha^−1^ for florpyrauxifen-benzyl, and 56.30625, 112.6125, 225.225, 450.45, 900.9, and 1801.8 g a.i. ha^−1^ for MCPA-Na. After the liquid on the stems and leaves had dried, the seedlings were placed in a greenhouse for cultivation. After 21 d, the aboveground grass was cut and weighed, and the inhibition rate was calculated. Each experimental treatment contained three biological replicates, and the experiment was conducted twice.

The effective rate of each herbicide causing 50% inhibition in plant height (GR_50_) was determined using the four-parameter logistic function with the “drc” add-on package [39] in the R 3.1.3 Language and Environment for Statistical Computing [40]. The model was defined as follows:

(1) Y=c+{(d−c)/(1+exp⁡(b(log x−log e)))}

The parameter e is also denoted GR_50_ and is the dose producing a response half-way between the upper limit, d, and the lower limit, c. The parameter b denotes the relative slope around e.

### 2.3. Construction of Chloroplast Genome

#### 2.3.1. DNA Sequencing and Genome Assembly

Total genomic DNA of *A. arenaria* (Aa1) and *A. multiflora* (Am1) was extracted using a modified cetyltrimethylammonium bromide method and applied to a 500 bp paired-end library construction using the NEBNext Ultra DNA Library Prep Kit (NEB, USA) for Illumina sequencing. Sequencing was performed on an Illumina NovaSeq 6000 platform (BerryGenomics Co., Ltd., Beijing, China). Approximately 4.6 and 5.7 GB of raw data from *A. arenaria* and *A. multifloras*, respectively, were generated with 150 bp paired-end read lengths. De novo assembly with NOVOPlasty (https://anaconda.org/bioconda/novoplasty/files?sort=ndownloads&sort_order=desc, accessed on 28 November 2022), and referencing the cp genome of closely related species, produced two options of circular contigs of the cp genome. The contig with the higher similarity to cpDNA was selected as the candidate cp genome. Several potential cp reads were extracted from the pool of Illumina reads using BLAST searches against the cp-genome results from NOVOPlasty and the related species *Rotala rotundifolia* (Accession number: NC_042888.1). Illumina cp reads were obtained to perform cp genome de novo assembly using the SPAdes-3.13.0 package (https://cab.spbu.ru/software/spades/, accessed on 28 November 2022). The NOVOPlasty assembly contig was optimized by the scaffolds from the SPAdes-3.13.0 result and aligned with the original clean Illumina reads using BWA, and the base correction was performed with Pilon v1.22. Finally, the assembled sequence was reordered and oriented according to the reference cp genome to generate the final assembled cp genomic sequence.

The MIcroSAtellite identification tool (http://webblast.ipk-gatersleben.de/misa/, accessed on 28 November 2022) was used for simple sequence repeat (SSR) analysis. The definitions (unit_size, min_repeats) were set to 1–10, 2–5, 3–4, 4–3, 5–3, and 6–3; the minimum distance between two SSRs was set to 100 bp. REPuter software (http://bibiserv.techfak.uni-bielefeld.de/reputer/, accessed on 28 November 2022) was used for long repeats (LR) analysis. The parameters were set as follows: minimal repeat size was 30 bp; the mismatch number, Hamming distance, was three; maximum computed repeats were 5000 (1 × 10^−3^).

#### 2.3.2. Genome Component Analysis and Gene Annotation

Genes encoding proteins, tRNAs, and rRNAs in the cp genomes of *A. arenaria* and *A. multifloras* were predicted using the GeSeq (https://chlorobox.mpimp-golm.mpg.de/geseq.html/, accessed on 28 November 2022) software. The specific parameters were set as follows: protein search identity: 60; rRNA, tRNA, DNA search identity: 35; third party tRNA annotators: tRNAscan-SE v2.0.7. High-accuracy gene bundles were obtained by removing the redundancy of predicted initial genes, followed by manual correction of the head, tail, and exon/intron boundaries of the genes. Finally, for the base composition of the cp genome, the gene distribution of each interval, including LSC, SSC, and IR, and the classification of each functional gene were counted and summarized. The protein sequences of cp genes were compared with known protein databases using BLASTP (https://ncbiinsights.ncbi.nlm.nih.gov/tag/blastp/, accessed on 28 November 2022) (evalue < 1 × 10^−5^.). Because there may have been more than one alignment result for each sequence, only one optimal alignment result was reserved as the database alignment information of the gene to ensure its biological significance. These databases included Non-Redundant Protein Sequence Database (NR) (http://www.ncbi.nlm.nih.gov/, accessed on 28 November 2022), Swiss-Prot (http://www.ebi.ac.uk/uniprot, accessed on 28 November 2022), Clusters of Orthologous Groups (COG), Kyoto Encyclopedia of Genes and Genomes (KEGG) (http://www.genome.jp/kegg/, accessed on 28 November 2022), and Gene Ontology (GO) (http://geneontology.org/, accessed on 28 November 2022). The amino acid sequences of *A. arenaria* and *A. multifloras* were aligned with the above databases to obtain functional annotation information for the coding genes.

### 2.4. Analysis of Genetic Relationship and Identification Characteristics

#### 2.4.1. Contraction and Expansion Analysis of Inverted Repeats Regions

We performed the IR contraction and expansion analysis for the two newly sequenced cp genomes of *A. arenaria* and *A. multiflora*. The four quadripartite structures (LSC, SSC, and two IR repeat regions) of each cp were compared, and changes in the copy number of related genes caused by contraction and expansion of the IR or pseudogenes resulting in boundary regions were analyzed. Genes that crossed or adjacent to the boundaries were obtained. In addition, the length and distance from the boundaries of these genes were analyzed.

#### 2.4.2. Phylogenetic Analysis

Eighteen cp genomes of plants, including the model plant of dicotyledon (*Arabidopsis thaliana*), rice field plants (*Eclipta prostrata* and *Persicaria lapathifolia*, Lythraceae plants (*Cuphea hyssopifolia*, *C. hookeriana*, *C. micropetala*, *Heimia apetala*, *H. myrtifolia*, *Pemphis acidula*, *Rotala rotundifolia*, *Woodfordia fruticose*, *Lagerstroemia subcostata*, *Lythrum salicaria*, and *Lawsonia inermis*), and Onagraceae plants (*Oenothera biennis*, *Ludwigia octovalvis*, *Epilobium hirsutum*, and *Circaea cordata*) which were downloaded from the NCBI database (accession numbers are shown in Supplementary Table S1), were selected for phylogenetic analysis with two *Ammannia* species. The sequences were aligned using ClustalW (v2.0.12) (http://www.clustal.org/clustal2/, accessed on 9 June 2023) with the default settings. The DNA substitution model was assessed using the Akaike information criterion [41]. The phylogenetic tree was constructed by the maximum likelihood (ML) method using PhyML v3.0 (htp://ww.atgc-montpeller. fr/phyml/, accessed on 9 June 2023), and bootstrap values were calculated for 1000 replicas [42,43]. The tree building model was finally evaluated using jModelTest 2.1.10 (https://github.com/ddarriba/jmodeltest2, accessed on 9 June 2023), with the best model “GTR + I + G”.

#### 2.4.3. Single Nucleotide Polymorphism (SNP) Analysis

Using MUMmer software (http://mummer.sourceforge.net/, accessed on 28 November 2022), the cp genome sequence of *A. multifloras* was completely aligned with the reference sequence, and the cp genome sequence of *A. arenaria* was used to identify sites with a difference between the two sets of cp genome sequences, perform preliminary filtering, and detect potential SNP sites. Sequences of 100 bp on both sides of the SNP site of the reference sequence were extracted and aligned with the assembly results using BLAT v35 software (http://hgdownload.soe.ucsc.edu/admin/exe/linux.x86_64/blat/, accessed on 28 November 2022) to verify the SNP site. If the alignment length was less than 101 bp, it was considered an unreliable SNP and was removed; if the alignment was repeated multiple times, the SNP was considered a repetitive region and was also removed, and reliable SNPs were obtained.

## 3. Results

### 3.1. Differences in Morphology and Seed Germination Characteristics

**Figure 1:**
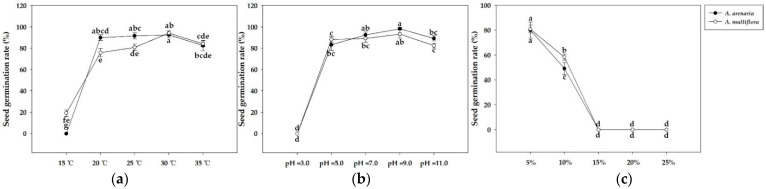
*Ammannia arenaria* and *A. multiflora* seed germination rate at different temperature, pH, and osmolarities. (**a**) The effect of temperature on germination. (**b**) The effect of pH on germination. (**c**) The effect of temperature on osmolarity. 5–25% represents the mass fraction of Macrogol 6000. “a–g” indicate significant differences (*p* < 0.05).

This study explored the differences in seeds germination conditions between *A. arenaria* and *A. multiflora* (Figure 1). The germination rates on the ninth day after treatment of *A. arenaria* seeds were 0, 90.00%, 91.67%, 92.50%, and 82.50% at 15, 20, 25, 30, and 35 °C, whereas the germination rates of *A. multiflora* seeds were 19.17%, 75.83%, 80.83%, 94.17%, and 84.17%, respectively. The germination rates of *A. arenaria* seeds were 0, 83.33%, 92.5%, 98.33%, and 89.17% at pH = 3.0, 5.0, 7.0, 9.0, and 11.0, whereas those of *A. multiflora* seeds were 0, 88.33%, 89.17%, 93.33%, and 82.50%, respectively. The germination rates of *A. arenaria* seeds were 80.00%, 49.17%, 0, 0, and 0 at osmolarity φ = −0.06, −0.17, −0.32, −0.53, and −0.79 Mpa, whereas those of *A. multiflora* seeds were 80.83%, 58.33%, 0, 0, and 0, respectively. This study also investigated the differences in the morphology of *A. arenaria* and *A. multiflora* during seed maturity in paddy fields (Table 2). The results showed that the plant height, maximum lateral distance, and seed dry weight quality of *A. arenaria* were significantly higher than those of *A. multiflora*. The frequency of occurrence of the two *Ammannia* species was very similar in the rice field.

### 3.2. Similar Sensitivity to Common Herbicides in Paddy Fields

**Figure 2:**
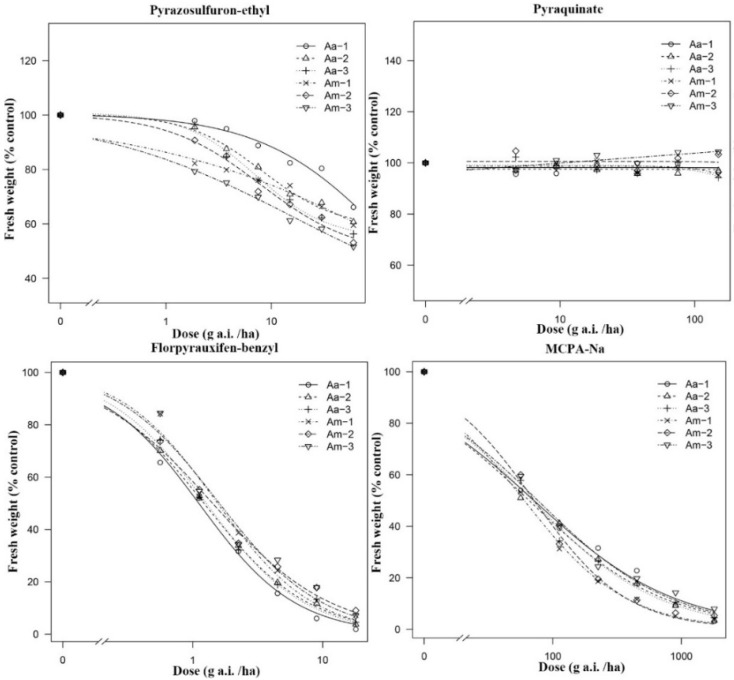
Dose–response analyses for response of *Ammannia arenaria* and *A. multiflora* to four herbicides, pyrazosulfuron-ethyl, pyraquinate, florpyrauxifen-benzyl, and MCPA-Na. The *X*-axis represents the dose (g a.i. ha^−1^). The *Y*-axis represents percentages of fresh weight (% untreated control). “Aa” means *A. arenaria*; “Am” means *A. multiflora*; “-1” means biotype 1; “-2” means biotype 2; “-3” means biotype 3.

The sensitivity of *A. arenaria* and *A. multiflora* to four herbicides, pyrazosulfuron-ethyl, pyraquinate, florpyrauxifen-benzyl, and MCPA-Na, were tested using the whole-plant bioassay (Figure 2). The GR_50_ of florpyrauxifen-benzyl to *A. arenaria* was 1.13 ± 0.09–1.26 ± 0.09 g a.i. ha^−1^ and the GR_90_ was 7.35 ± 0.91–9.34 ± 1.26 g a.i. ha^−1^. The GR_50_ of florpyrauxifen-benzyl to *A. multiflora* was 1.40 ± 0.12–1.54 ± 0.12 g a.i. ha^−1^ and the GR_90_ was 10.04 ± 1.28–14.26 ± 2.21 g a.i. ha^−1^. The GR_50_ of MCPA-Na to *A. arenaria* was 65.80 ± 6.52–71.80 ± 7.17 g a.i. ha^−1^ and the GR_90_ was 826.13 ± 122.47–1158.02 ± 178.10 g a.i. ha^−1^. The GR_50_ of MCPA-Na to *A. multiflora* was 59.89 ± 4.82–75.89 ± 6.90 g a.i. ha^−1^ and the GR_90_ was 442.60 ± 55.24–1043.48 ± 165.68 g a.i. ha^−1^, respectively. Pyrazosulfuron-ethyl could not inhibit the fresh weight inhibition rate of *A. arenaria* and *A. multiflora* to above 80% under indoor conditions. Pyraquinate was ineffective in causing any interference with the fresh weight of the two plants.

### 3.3. Differences in Chloroplast Genome Composition

#### 3.3.1. Chloroplast Genome Features

**Figure 3:**
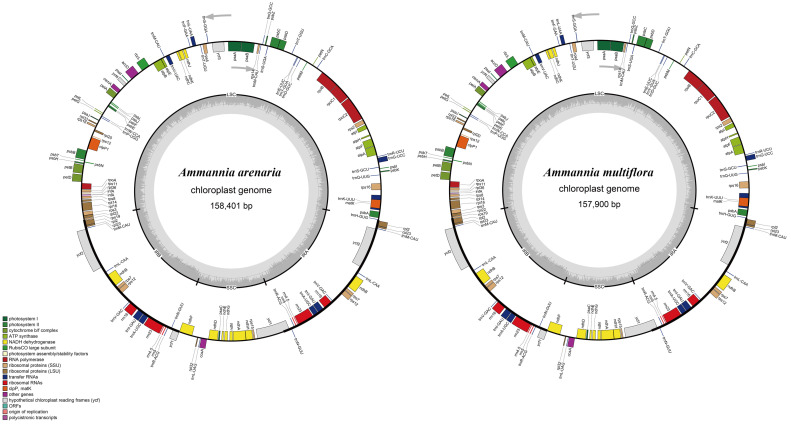
Assembly, size, and features of cp genomes of *Ammannia arenaria* and *A. multiflora*. The genes outside the circle are transcribed in the counterclockwise direction, and the genes inside the circle are transcribed in the clockwise direction. Different colors in genes represent different functions. The dark gray area and light gray area of the inner circle represent the GC content to AT content of the genome, respectively.

The cp genome libraries of *A. arenaria* and *A. multiflora* were constructed, and raw reads were deposited in the NCBI GenBank database (accession number: PRJNA904652 and PRJNA904683). The complete cp genome sequences of *A. arenaria* and *A. multiflora* are 158,401 and 157,900 bp in length, respectively, with both having an evident quadripartite structure, including LSC, SSC, and a pair of IRs (IRa and IRb) (Figure 3). The lengths of LSC, SSC, and IRs were 88,911, 17,954, and 25,768 bp in *A. arenaria*, and 88,410, 17,954, and 25,768 bp in *A. multiflora*, respectively, and there were slight differences in GC content between individual structures of the two cp genomes (Table 3). The cp genome of *A. arenaria* contained 86 protein-coding genes, and that of *A. multiflora* contained 85 protein-coding genes (Table 3). Each cp genome contained 37 transfer RNA (tRNA) and eight ribosomal RNAs (rRNA) genes (Table 3). There were 72 (71 in *A. multiflora*) protein-coding and 26 tRNA genes located within the LSC; 10 (IRb) or nine (IRa) protein-coding, nine tRNA-coding, and four rRNA-coding genes located within IR; and 14 protein-coding and one tRNA genes located within the SSC (Figure 3).

#### 3.3.2. Sequence Repeats

A total of 91 and 90 simple sequence repeats (SSRs) were identified in *A. arenaria* and *A. multiflora* cp genomes, respectively. Five were on IRa, five were on IRb, 65 were on LSC, and 16 were on SSC in *A. arenaria*, whereas five were on IRa, five were on IRb, 66 were on LSC, and 14 were on SSC in *A. multiflora*. Additionally, only 15 and 14 SSRs were distributed in the coding region in the cp genomes of *A. arenaria* and *A. multiflora*, respectively. (Table 4). The number of nucleotides of all SSRs is fewer than or equal to four. Mononucleotide repeats were the most abundant SSRs, accounting for 75.8% and 76.6% of the total SSRs in cp genomes of *A. arenaria* and *A. multiflora*, of which 68 repeat units were A/T, and only one was G (Supplementary Table S2). There were 127 long repeats (LR) in the cp genome of *A. arenaria*, including 23 with a Hamming distance (HD) = 0, 14 with HD = 1, 25 with HD = 2, and 65 with HD = 3, whereas in the cp genome of *A. multiflora*, there were 99 LRs, including six with HD = 0, 10 with HD = 1, 22 with HD = 2, and 61 with HD = 3 (Table 4). In the cp genomes of *A. arenaria* and *A. multiflora*, the frequency of forward and palindromic repetition sequences was the highest (accounting for 85.8% and 96.0%). Most LRs were distributed on the hydrodynamic cp open reading frame 2 (*ycf2*) genes (Supplementary Tables S3 and S4).

#### 3.3.3. Gene Annotation and Classification

**Figure 4:**
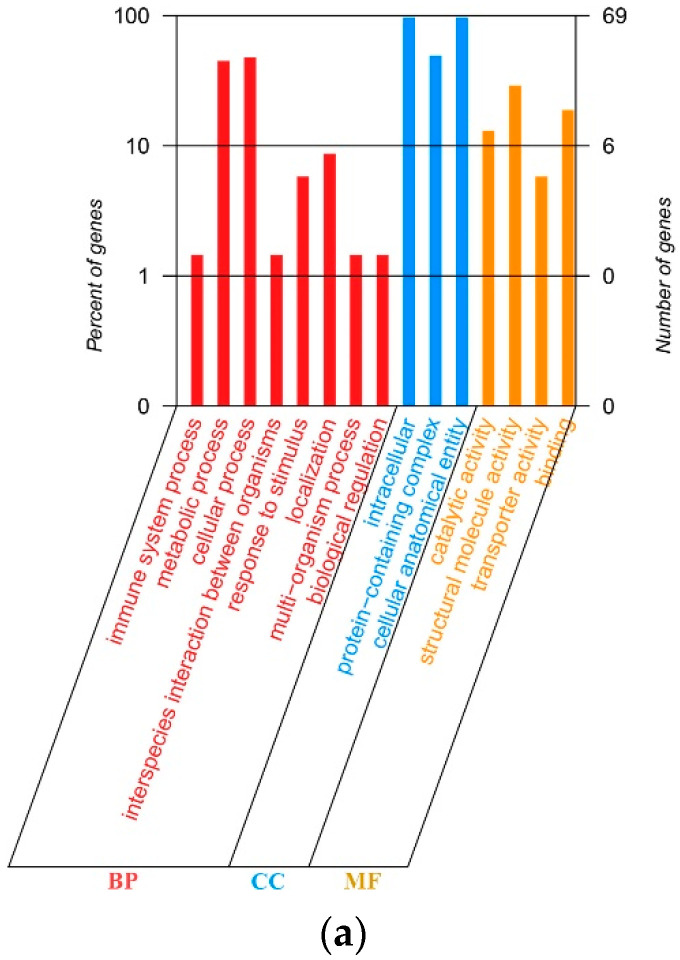

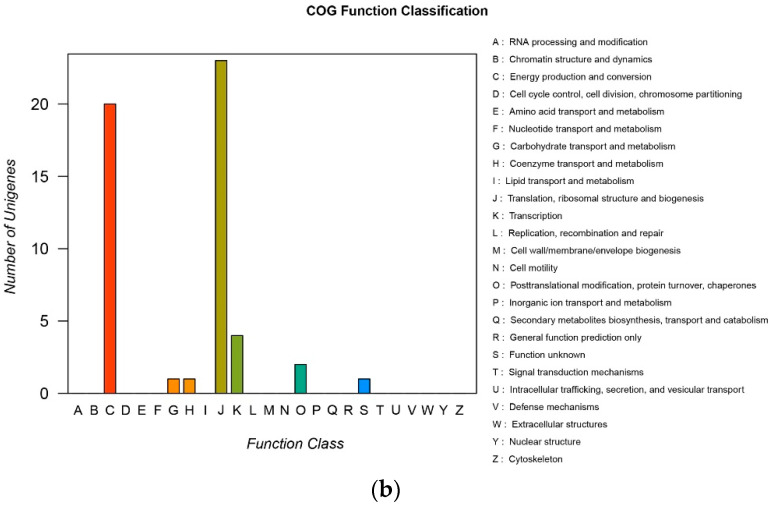
Classifications of genes function of *Ammannia arenaria* and *A. multiflora*. (**a**) Percentages of genes matched to GO function classification. BP means biological process, CC means cellular component, and MF means molecular function. (**b**) Number of unigenes matched to COG function classification.

All protein-encoding genes in the cp genomes of *A. arenaria* and *A. multiflora* were functionally annotated and mainly belonged to the photosynthesis and self-replication categories. The gene names, groups, and categories are listed in Table 5. Compared to *A. multiflora*, the cp genome of *A. arenaria* has one unique gene, *infA* (Table 5 and Figure 3). In the two cp genomes, 82 genes were matched to the Non-Redundant Protein Sequence Database (NR), 69 to Gene Ontology (GO), 54 to Clusters of Orthologous Groups (COG), 73 to Kyoto Encyclopedia of Genes and Genomes (KEGG), and 84 to Swiss. Among these genes, 45 were matched to all five databases in the two *Ammannia* species; 84 to at least one database in the species; the unmatched genes were *psbL* and *infA* in *A. arenaria* and *psbL* in *A. multiflora*. Genes matched to GO were further classified as the biological process (BP), cellular component (CC), and molecular function (MF), with most genes classified as BP (Figure 4a). Genes matched to KEGG were mainly involved in energy production and conversion, translocation, ribosomal structure and biogenesis, and transcription pathways (Figure 4b).

### 3.4. Genetic Affinity and Differences

#### 3.4.1. IR Expansion and Contraction

**Figure 5:**
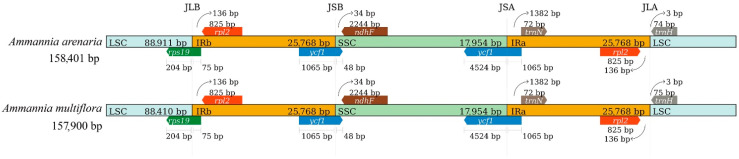
Comparison of LSC, IRb, SSC, and IRa border regions in two species of *Ammannia* species.

To further observe the potential expansion and contraction of the IR regions, gene variations at the IR/SSC and IR/LSC boundary regions of two *Ammannia* species were compared (Figure 5). The gene *rps19* crosses the junction of LSC and IRb, with 204 bp in the LSC region and 75 bp in the IRb region. The gene *rpl2*, located in IRb, is a boundary gene, 136 bp away from the LSC region. The gene *ycf1* crosses the junction of the IRb and SSC regions, with 1065 bp in the IRb region and 48 bp in the SSC region. The gene *ndhF*, located in the SSC region, is a boundary gene, 34 bp away from the IRb region. The gene *ycf1* crosses the junction of the SSC and IRa regions, with 4524 bp in the SSC region and 1065 bp in the IRa region. The gene *trnN*, located in the IRa region, is a boundary gene, 1382 bp away from the SSC region. The gene *rpl2*, located in the IRa region, is a boundary gene, 136 bp away from the LSC region. The gene *trnH*, located in the LSC region, is a boundary gene, 3 bp away from the IRa region. Except for *trnH*, the lengths of the other boundary genes were consistent in *A. arenaria* and *A. multiflora*. The length of *trnH* is 74 bp in *A. arenaria* and 75 bp in *A. multiflora*.

#### 3.4.2. Phylogenetic Tree

**Figure 6:**
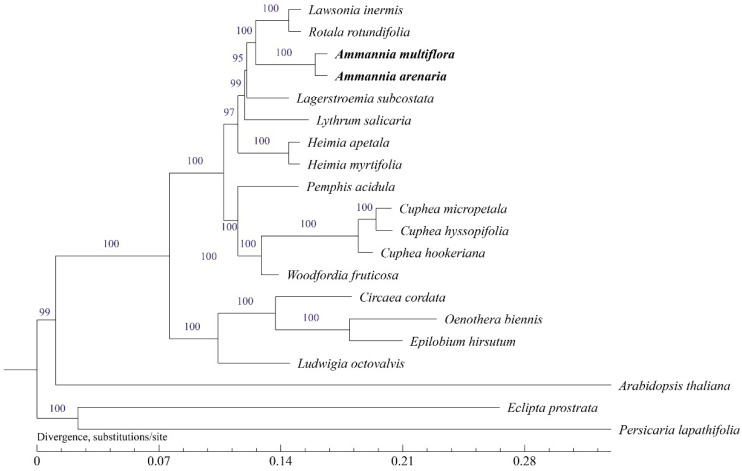
Phylogenetic tree for 20 species using maximum likelihood, based on alignments of complete chloroplast genomes. The numbers at the nodes indicate bootstrap values from 1000 replicates.

Phylogenetic trees were generated using maximum likelihood (ML) and Bayesian inference (BI) analysis methods based on 20 complete cp genomes showing the same topology (Figure 6). In this study, the representative plants of each genus of Lythraceae are located on one branch, indicating their close relationship. Two *Ammannia* plants clustered into a single clade formed a monophyletic group with high support (support value (BS) = 100 for ML). *R. rotundifolia* and *L. inermis* were the closest relative to two *Ammannia* species (BS = 100 for ML), and they had the most recent common ancestor (MRCA) with *L. subcostata* (BS = 95)*. L. salicaria*, *H. apetala*, and *H. myrtifolia* also had relatively close genetic relationships with the two *Ammannia* species. The other five Lythraceae plants were located on another branch. *O. biennis*, *L. octovalvis*, *E. hirsutum*, and *C. cordata*, belonging to the sister family, Onagraceae, had a close relationship with Lythraceae. The two *Ammannia* species are not closely related to the model plant, *A. thaliana*. Additionally, the two *Ammannia* species and another two common dicotyledonous weeds in rice fields, *E. prostrata* and *P. lapathifolia*, had the more distant affix relationship.

#### 3.4.3. Single Nucleotide Polymorphism

SNP analysis was performed to further explore the DNA sequence polymorphisms and differences caused by single nucleotide variation in *A. arenaria* and *A. multiflora* (Table 6). It indicated that 67 SNPs were detected in the cp genome of *A. arenaria* compared to *A. multiflora*, representing 47 (70.15%) in intergenic regions and 20 (29.85%) in CDS regions. No mutations appeared in the start and stop codons. There were 11 synonymous (16.42%) and nine nonsynonymous (13.43%) mutations in 13 genes, including *ndhD*, *ycf1*, *ccsA*, *atpA*, *psaB*, *psbB*, *psbM*, *rpl20*, *rpl32*, *rpoB*, *rpoC2*, *rps8*, and *ndhA*. Among them, four SNPs were found in *adhD* and *ycf1*, which was the most among all the genes found to have SNPs, and three nonsynonymous SNPs were found in *ycf1*, which was the most among all the genes found to have nonsynonymous SNPs (Supplementary Table S5). The locations of all SNPs of the cp genome of *A. arenaria* were shown in Supplementary Table S6.

## 4. Discussion

*Ammannia* species, *A. arenaria* and *A. multiflora*, are the most common broad-leaved weeds in paddy fields in China. Although farmers use various methods for weed management, situations may still exist where *Ammannia* species are uncontrollable (Supplementary Figure S2). The conditions required for seed germination of the two *Ammannia* species were similar (Figure 1). However, *A. multiflora* seeds can germinate at 15 °C. This should be taken seriously when planting early rice. Therefore, the results can provide a theoretical basis for predicting the occurrence of two weeds under different environmental conditions. Based on our investigation, the height and maximum lateral distance of *A. arenaria* were higher than *A. multiflora* (Table 2), indicating that *A. arenaria* has a considerable advantage in competing for resources with rice. Therefore, identifying and managing *A. arenaria* in the early stages is particularly important. Simultaneously, we should also be alert to the risk of future damage to rice from plants closely related to *Ammannia* species.

We selected three populations for each species to avoid the impact of herbicide use history on the study of sensitivity differences between *A. arenaria* and *A. multiflora*. The synthetic auxin herbicides, florpyrauxifen-benzyl [44] and MCPA-Na [45], had the best control effect on the two *Ammannia* species; however, the traditional acetolactate synthase inhibitor, pyrazosulfuron-ethyl [46], and the new 4-hydroxyphenylpyruvate dioxygenase inhibitor, pyraquinate [47], were ineffective in managing them. This study can serve as a basis for herbicide selection. Accurately identifying and managing *Ammannia* species can also help reduce herbicide costs and environmental pollution.

Many plant cp genome sequences have been determined following the first reported cp genome sequence of tobacco [48]. Presently, there are no studies on the evolutionary relationships of *Ammannia*. The present study found that the cp genomes of *A. arenaria* and *A. multiflora*, 158,401 and 157,900 bp (Figure 3), were relatively larger than those of common plants, such as *Echinochloa* and *Oryza*, and smaller than those of *Cyperus* species in paddy fields [49]. The typical circular tetramerous structure of the cp genome is conserved in plants, and the length of each quadripartite structure of the cp genome in the same genus is generally similar [37,50]. The cp genome of *A. arenaria* and *A. multiflora* also revealed these features, with similar LSC, SSC, and IR lengths (Figure 3; Table 3).

Simple sequence repeats, or microsatellites, are tandem repeats comprising 1–6 nucleotide repeat units that are widely distributed in plant cp genomes [51,52]. As valuable molecular genetic markers, SSRs are widely used in plant genotyping and population genetics [53,54,55,56]. These repeats promote intermolecular recombination and enrich the diversity of cp genomes in the population [57]. This study showed that the cp genome of *A. arenaria* had one more SSR than that of *A. multiflora*, including one SSR with an encoding function. Thus, differential SSRs can be used as important molecular markers in the two species. Additionally, long repeats are special DNA sequences that are repeated in the genome in various forms and usually occupy a large proportion of the genome [58]. Repeated segments also have important molecular significance in the study of plant evolution [59]. The cp genome of *A. arenaria* had 28 more LRs than that of the cp genome of *A. multiflora* (Table 4). The repeat sequences detected in this study are important biological information resources for *Ammannia*, and are of considerable significance for the identification of *Ammannia* species and the study of genetic diversity and population structure.

Chloroplast genome genes are highly conserved in plants [24,25,26]. As a result, 86 and 85 protein-coding genes were identified in *A. arenaria* and *A. multiflora*, respectively. Although the genes were not completely consistent, the categories of genes were similar, mainly belonging to the categories of photosynthesis and self-replication (Table 5), further verifying the conservation of protein-encoding genes in chloroplasts [27,50,60]. The difference in the number of protein-coding genes between the two *Ammannia* species is caused by one gene, *infA*, which exists only in the cp genome of *A. arenaria* (Figure 3; Table 5). The *infA* gene is a ribosomal protein L23 operon component and is transcribed into polycistronic mRNA [61]. The *infA* gene is considered to be the most mobile chloroplast gene in plants so far [62], which may have caused the difference between *A. arenaria* and *A. multiflora* in evolution. The *infA* gene in *A. arenaria* had an initiation codon, unlike without an initiation codon in tobacco [48]. Additionally, this different gene can be used to distinguish between the morphologically-similar *A. arenaria* and *A. multiflora*. Except for protein-coding genes, noncoding RNAs are conservative in the two *Ammannia* species, similar to other plants of the same genus [27,60].

Expansion and contraction of the cp genome is a common phenomenon in plants [24], which occurs mainly at the IR/SC junction [63]. Although highly conserved, IR expansion and contraction are directly related to cp genome rearrangement and variation in size, which is also a major determining factor in plant genome evolution [27,33,37]. This study showed that the IR expansion and contraction of the cp genome were highly conserved between *A. arenaria* and *A. multiflora*. All boundary genes or genes that cross two regions are consistent in the two *Ammannia* species, including the length of these genes away from the nearest boundary. There was a difference in the length of only one gene, *trnH*, between *A. arenaria* and *A. multiflora*, which was 74 and 75 bp, respectively (Figure 5). This revealed that the expansion and contraction in the IR and SC regions did not result in large changes to the junction boundaries in *Ammannia*.

Genome data are valuable for addressing species definitions, as they can be used to establish organelle-based “barcodes” for certain species, which can be used to reveal phylogenetic relationships [64]. Chloroplast genome sequences are essential for plant species identification, phylogenetic relationships, and the determination of plant taxonomic status. With the continuous discovery of plant cp genome information, the genetic evolutionary relationships of some Lythraceae plants have been successfully elucidated in the form of phylogenetic trees [65,66,67]. However, the phylogenetic relationships of *Ammannia* have not yet been studied. In the present study, the two cp genomes of *Ammannia*, model plant of dicotyledon (*A. thaliana*), common dicotyledonous weeds in rice field, Lythraceae plants, and Onagraceae plants were used to perform phylogenetic analysis. The analysis showed that the morphologically-similar *Ammannia* species, *A. arenaria* and *A. multiflora*, were close phylogenetically (Figure 6). Thirteen Lythraceae plants, including *A. arenaria* and *A. multiflora*, are more closely related, with support values of 100%, while Lythraceae and Onagraceae have a sister relationship, which is consistent with previous research results [66]. However, the genetic relationship between *Ammannia* species and another two dicotyledonous weeds in rice fields, *E. prostrata* and *P. lapathifolia*, was distant. Although analysis of the complete cp genome may not be sufficient to adequately resolve all phylogenetic relationships [68,69,70], it still provides a viable way to clarify species relationships.

Single nucleotide polymorphisms are important indicators of evolutionary differences between plants of the same genus, with the advantage of low cost by high-throughput techniques [71]. These direct molecular markers evidently show the exact nature and location of allelic variations [72]. Therefore, SNPs have recently attracted increasing attention [33,53]. Considering the cp genome of *A. multiflora* as a reference, 47 SNPs in the intergenic region and 20 SNPs in the CDS region were identified in *A. arenaria* (Table 6), showing the difference between the two species. This is one of the important molecular foundations for the differentiation of two species. The nine nonsynonymous SNPs may result in the differences in protein function. These SNPs can be important differential nucleotide databases to distinguish the two species. Generally, SNPs occur at a higher frequency in variable, less conserved genes [72]. The present study identified nine nonsynonymous SNPs across six encoding genes (Supplementary Table S5), accounting for only approximately 7% of all genes in the cp genome of *A. arenaria*. This is because the nonsynonymous rate is typically slower owing to the purifying selection acting on the gene [73].

## 5. Conclusions

The cp genomes of *A. arenaria* and *A. multifloras* were first sequenced, revealing a close relationship in our study. Although the two Ammannia species are very similar in morphology at the seedling stage in paddy fields, some differences exist in their cp genomes. These differences were mainly reflected in the genome length, protein-coding genes, and SNPs. Although we speculated that the differentiation time of *A. arenaria* and *A. multifloras* was relatively short, the results of the IR expansion and contraction and the phylogenetic tree revealed differences in the evolutionary directions of *A. arenaria* and *A. multifloras*, which is the molecular basis of biodiversity. Our results provide important biological information for the identification and evolution of *Ammannia*.

## Figures and Tables

**Table 1:** Information on seed collection locations for *Ammannia arenaria* and *A. multiflora*.

Species	Population	Collection Sites	Latitude and Longitude
*A. arenaria*	Aa-1	Zhongjiu Village, Pudong District, Shanghai	30.93° N, 121.87° E
	Aa-2	Wanhong Village, Pudong District, Shanghai	30.98° N, 121.82° E
	Aa-3	Panghuang Village, Chongming District, Shanghai	31.56° N, 121.68° E
*A. multiflora*	Am-1	Shenlian Village, Qingpu District, Shanghai	31.24° N, 121.14° E
	Am-2	Qiaobei Village, Pudong District, Shanghai	31.02° N, 121.81° E
	Am-3	Huaxi Village, Chongming District, Shanghai	31.79° N, 121.20° E

**Table 2:** Differences in morphology of *Ammannia arenaria* and *A. multiflora* in paddy fields.

Biological Morphology Features	*A. arenaria*	*A. multiflora*
Height (cm)	109.19 ± 1.55	81.03 ± 2.70 *
Maximum lateral distance (cm)	35.20 ± 0.71	15.23 ± 1.26 *
Weight of a thousand seed (g)	0.0220 ± 0.0001	0.0180 ± 0.0001 *
Frequency of occurrence (%)	19.1 ± 3.3	19.0 ± 3.8

The symbol “*” means significant differences between two species according to Student’s *t*-test (*p* ≤ 0.05).

**Table 3:** Summary of *Ammannia arenaria* and *A. multiflora* chloroplast genome features.

Genome Features	*A. arenaria*	*A. multiflora*
Genome size (bp)	158,401	157,900
LSC length (bp)	88,911	88,410
SSC length (bp)	17,954	17,954
IR length (bp)	25,768	25,768
Protein-coding genes (bp)	80,028	79,908
Intergenic region length (bp)	78,373	77,992
Overall GC content (%)	36.73	36.73
GC content of LSC (%)	34.64	34.63
GC content of SSC (%)	30.61	30.60
GC content of IR (%)	42.46	42.46
Gene’s GC content (%)	37.39	37.42
Number of protein-coding genes	86	85
Number of tRNA	37	37
Total length of tRNA (bp)	2835	2836
Number of rRNA	8	8
Total length of rRNA (bp)	9048	9048

**Table 4:** Simple sequence repeats (SSR) and long repeats (LR) in chloroplast genome of *Ammannia arenaria* and *A. multiflora*.

	Region/Hamming Distance	*A. arenaria*	*A. multiflora*
SSR	Coding	15	14
	Genome	91	90
	IRa	5	5
	IRb	5	5
	LSC	65	66
	SSC	16	14
LR	0	23	6
	1	14	10
	2	25	22
	3	65	61
	Total	127	99

**Table 5:** List of genes encoded by the chloroplast genome of *Ammannia arenaria* and *A. multiflora*.

Category	Groups	Genes
Photosynthesis	Subunits_of_photosystem_I	*psaA*, *psaB*, *psaC*, *psaI*, *psaJ*
	Subunits_of_photosystem_II	*psbA*, *psbB*, *psbC*, *psbD*, *psbE*, *psbF*, *psbH*, *psbI*, *psbJ*, *psbK*, *psbL*, *psbM*, *psbN*, *psbT*, *psbZ*
	Subunits_of_NADH_dehydrogenase	*ndhA*, *ndhB*, *ndhB*, *ndhC*, *ndhD*, *ndhE*, *ndhF*, *ndhG*, *ndhH*, *ndhI*, *ndhJ*, *ndhK*
	Subunits_of_cytochrome_b/f_complex	*petA*, *petB*, *petD*, *petG*, *petL*, *petN*
	Subunits_of_ATP_synthase	*atpA*, *atpB*, *atpE*, *atpF*, *atpH*, *atpI*
	Large_subunit_of_Rubisco	*rbcL*
Self-replication	Large_subunits_of_ribosome	*rpl14*, *rpl16*, *rpl2* (×2), *rpl20*, *rpl22*, *rpl23* (×2), *rpl32*, *rpl33*, *rpl36*
	Small_subunits_of_ribosome	*rps11*, *rps12* (×2), *rps14*, *rps15*, *rps16*, *rps18*, *rps19*, *rps2*, *rps3*, *rps4*, *rps7* (×2), *rps8*
	DNA-dependent_RNA_polymerase	*rpoA*, *rpoB*, *rpoC1*, *rpoC2*
	Ribosomal_RNAs	*rrn16*, *rrn23*, *rrn4.5*, *rrn5*
	Transfer_RNAs	37 tRNAs
Other genes	Maturase	*matK*
	Protease	*clpP1*
	Envelope_membrane_protein	*cemA*
	Acetyl-CoA_carboxylase accD	
	C-type_cytochrome_synthesis_gene	*ccsA*
	Translation_initiation_factor	*infA* **(only in *A. arenaria*)**
	protochlorophillide_reductase_subunit	
Genes of unknown function	Proteins_of_unknown_function	*ycf1* (×2), *ycf2* (×2), *ycf3*, *ycf4*

**Table 6:** Nucleotide polymorphisms (SNPs) in the chloroplast genome of *Ammannia arenaria* compared to *A. multiflora*.

Mutate Type	Start	Stop	Synonymous	Nonsynonymous	CDS	Intergenic	Total_SNP
SNP Number	0	0	11	9	20	47	67
SNP Percentage (%)	0	0	16.42	13.43	29.85	70.15	100.00

## Data Availability

Raw reads of cp genomes of *Ammannia arenaria* and *A. multifloras* were deposited in the NCBI GenBank database (accession number: PRJNA904652 and PRJNA904683).

## Supplementary Materials

The following supporting information can be downloaded at: https://www.mdpi.com/article/10.3390/biology12070936/s1, Figure S1: Morphology of Ammannia arenaria and A. multiflora; Figure S2: Damage of Ammannia species to rice; Table S1: Accession numbers of plants downloaded from the NCBI database for phylogenetic analysis; Table S2: SSR statistic in the chloroplast genomes of Ammannia arenaria and A. multifloras; Table S3: Long Repeat in the chloroplast genome of Ammannia arenaria; Table S4: Long Repeat in the chloroplast genome of Ammannia multiflora; Table S5: SNPs in CDS region of the chloroplast genome of Ammannia arenaria; Table S6: Distribution of all SNPs of the chloroplast genome of Ammannia arenaria.
