# Supplementary figures and images for: Comparison of Biological and Genetic Characteristics between Two Most Common Broad-Leaved Weeds in Paddy Fields: Ammannia arenaria and A. multiflora (Lythraceae)
Source: Biology (Basel). 2023 Jun 30;12(7):936. doi: 10.3390/biology12070936 (PMC10375975; doi:10.3390/biology12070936)

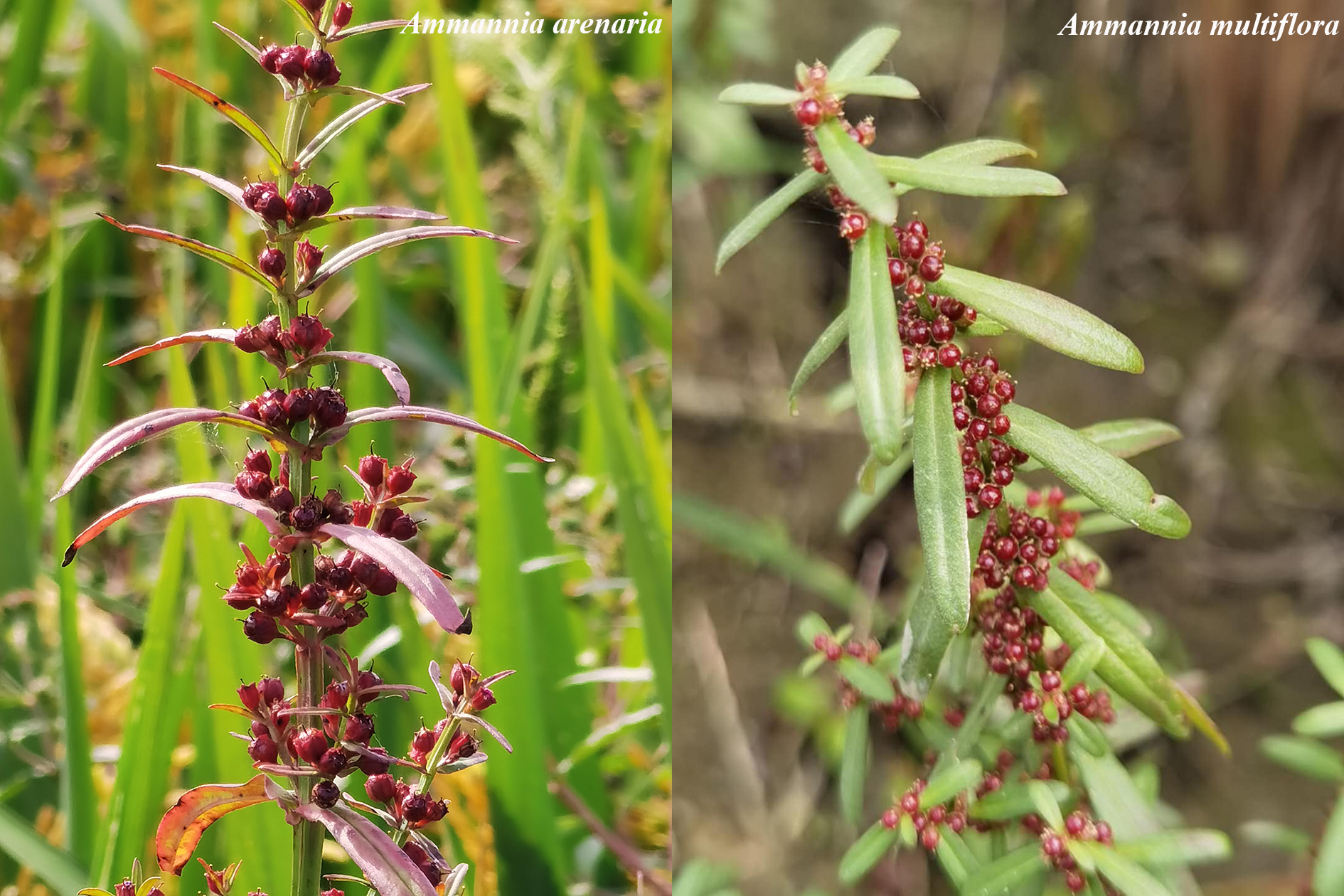

Supplement: Supplementary file 1 [file biology-12-00936-s001.zip › Supplementary Figure S1. Morphology of Ammannia arenaria and A. multiflora.jpg]

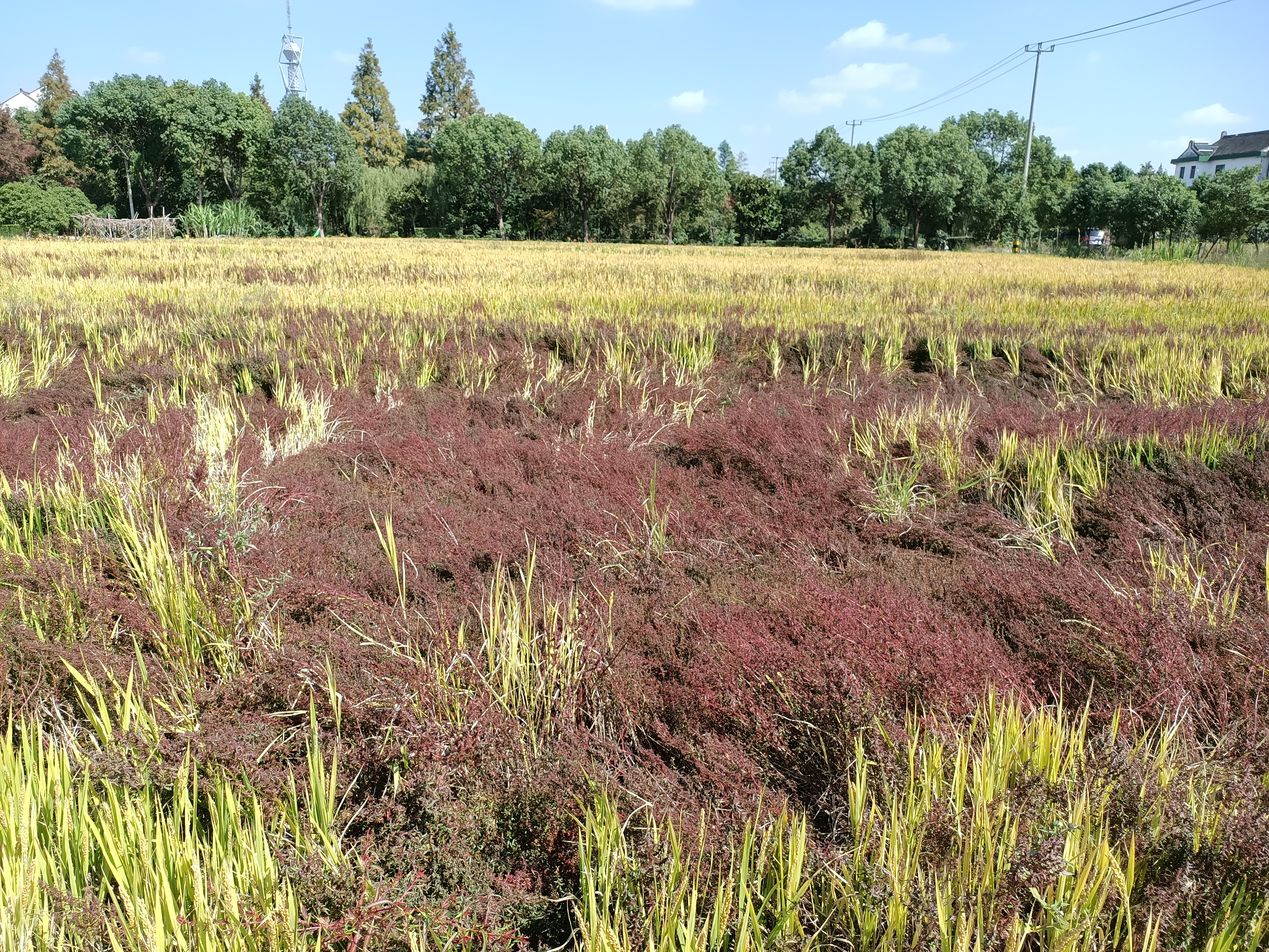

Supplement: Supplementary file 1 [file biology-12-00936-s001.zip › Supplementary Figure S2. Damage of Ammannia species to Rice.jpg]
